# Rationale and design of interleukin-1 blockade in recently decompensated heart failure (REDHART2): a randomized, double blind, placebo controlled, single center, phase 2 study

**DOI:** 10.1186/s12967-022-03466-9

**Published:** 2022-06-15

**Authors:** Benjamin Van Tassell, Virginia Mihalick, Georgia Thomas, Amr Marawan, Azita H. Talasaz, Juan Lu, Le Kang, Amy Ladd, Juan Ignacio Damonte, Dave L. Dixon, Roshanak Markley, Jeremy Turlington, Emily Federmann, Marco Giuseppe Del Buono, Giuseppe Biondi-Zoccai, Justin M. Canada, Ross Arena, Antonio Abbate

**Affiliations:** 1 Pauley Heart Center, Department of Internal Medicine, Virginia Commonwealth University, Pauley Heart Center, Richmond, VA USA; 2 Department of Pharmacotherapy and Outcome Sciences, Virginia Commonwealth University, Richmond, VA USA; 3 Division of Epidemiology, Department of Family Medicine and Population Health, Virginia Commonwealth University, Richmond, VA USA; 4 Department of Biostatistics, Virginia Commonwealth University, Richmond, VA USA; 5 Department of Medical-Surgical Sciences and Biotechnologies, Sapienza University of Rome, Latina, Italy; 6 Mediterranea Cardiocentro, Naples, Italy; 7 Department of Physical Therapy, College of Applied Science, University of Illinois Chicago, Chicago, IL USA

**Keywords:** IL-1, Interleukin-1, Heart failure, Anakinra, Treatment, Target therapy, HFrEF

## Abstract

**Background:**

Heart failure (HF) is a global leading cause of mortality despite implementation of guideline directed therapy which warrants a need for novel treatment strategies. Proof-of-concept clinical trials of anakinra, a recombinant human Interleukin-1 (IL-1) receptor antagonist, have shown promising results in patients with HF.

**Method:**

We designed a single center, randomized, placebo controlled, double-blind phase II randomized clinical trial. One hundred and two adult patients hospitalized within 2 weeks of discharge due to acute decompensated HF with reduced ejection fraction (HFrEF) and systemic inflammation (high sensitivity of C-reactive protein > 2 mg/L) will be randomized in 2:1 ratio to receive anakinra or placebo for 24 weeks. The primary objective is to determine the effect of anakinra on peak oxygen consumption (VO_2_) measured at cardiopulmonary exercise testing (CPX) after 24 weeks of treatment, with placebo-corrected changes in peak VO_2_ at CPX after 24 weeks (or longest available follow up). Secondary exploratory endpoints will assess the effects of anakinra on additional CPX parameters, structural and functional echocardiographic data, noninvasive hemodynamic, quality of life questionnaires, biomarkers, and HF outcomes.

**Discussion:**

The current trial will assess the effects of IL-1 blockade with anakinra for 24 weeks on cardiorespiratory fitness in patients with recent hospitalization due to acute decompensated HFrEF.

Trial registration: The trial was registered prospectively with ClinicalTrials.gov on Jan 8, 2019, identifier NCT03797001.

## Introduction

Heart failure (HF) represents a leading cause of morbidity and mortality worldwide, despite improvements in treatments and widespread efforts to implement guideline directed medical therapies [1, 2]. The high morbidity and mortality rates of patients with HF highlights the urgent need to identify new axes of disease pathogenesis for the development of novel therapies.

There is a mutual interplay between HF and inflammation suggesting that inflammation contributes to the pathogenesis and progression of HF [3–8]. Inflammation is highly prevalent in patients with HF, correlates with disease severity and appears to be more pronounced in patients with acute HF [9–12]. Comorbidities as well as acute stressors (i.e., ischemia, increased myocardial wall tension, hemodynamic overload) lead to the activation of the inflammatory response in the heart with activation of the NOD-, LRR- and pyrin domain-containing protein 3 (NLRP3) inflammasome, subsequent maturation and release of proinflammatory cytokines such as Interleukin-1beta (IL-1β) and IL-18 [13]. IL-1β is one of two IL-1 isoforms shown to function as a cardio-depressant factor, and to promote adverse cardiac remodeling after myocardial injury [14–17]. IL-1 blockade with anakinra has been shown to prevent adverse cardiac remodeling and HF in animal models of acute myocardial infarction (AMI) and reduce the incidence of HF in patients with ST-segment elevation AMI [18, 19]. In the Canakinumab Anti-inflammatory Thrombosis Outcome Study (CANTOS) trial, canakinumab, an IL-1β antibody, led to a significant reduction of the composite of HF hospitalization or HF–related mortality in a population of 10,061 patients with prior AMI and residual inflammatory burden [20]. In the REcently Decompensated Heart failure Anakinra Response Trial (REDHART) study, anakinra treatment led to an improvement in peak oxygen consumption (VO_2_), a primary measure of aerobic exercise capacity and cardiorespiratory fitness (CRF) [21, 22], in the group treated for 12 weeks, but not in the group treated for 2 weeks [23].

The REDHART2 study is designed to expand the previous findings, determine the effects of anakinra treatment administered for 24 weeks on peak VO_2_ and other measures of CRF (CRF is considered a vital sign) as well as derive an estimate for the potential effect size on HF outcomes[21].

### Study objectives and hypothesis

The objective of the study is to determine the effects of IL-1 blockade with anakinra on peak VO_2_ (i.e., primary outcome) derived from cardiopulmonary exercise testing (CPX) after 24 weeks of treatment, as well as the effects of anakinra on cardiac biomarkers, echocardiographic data, noninvasive hemodynamic, body composition analysis, quality of life (QoL) questionnaires, perceived functional capacity and physical activity questionnaires, and HF-related clinical outcomes.

## Methods

### Study design

**Fig. 1:**
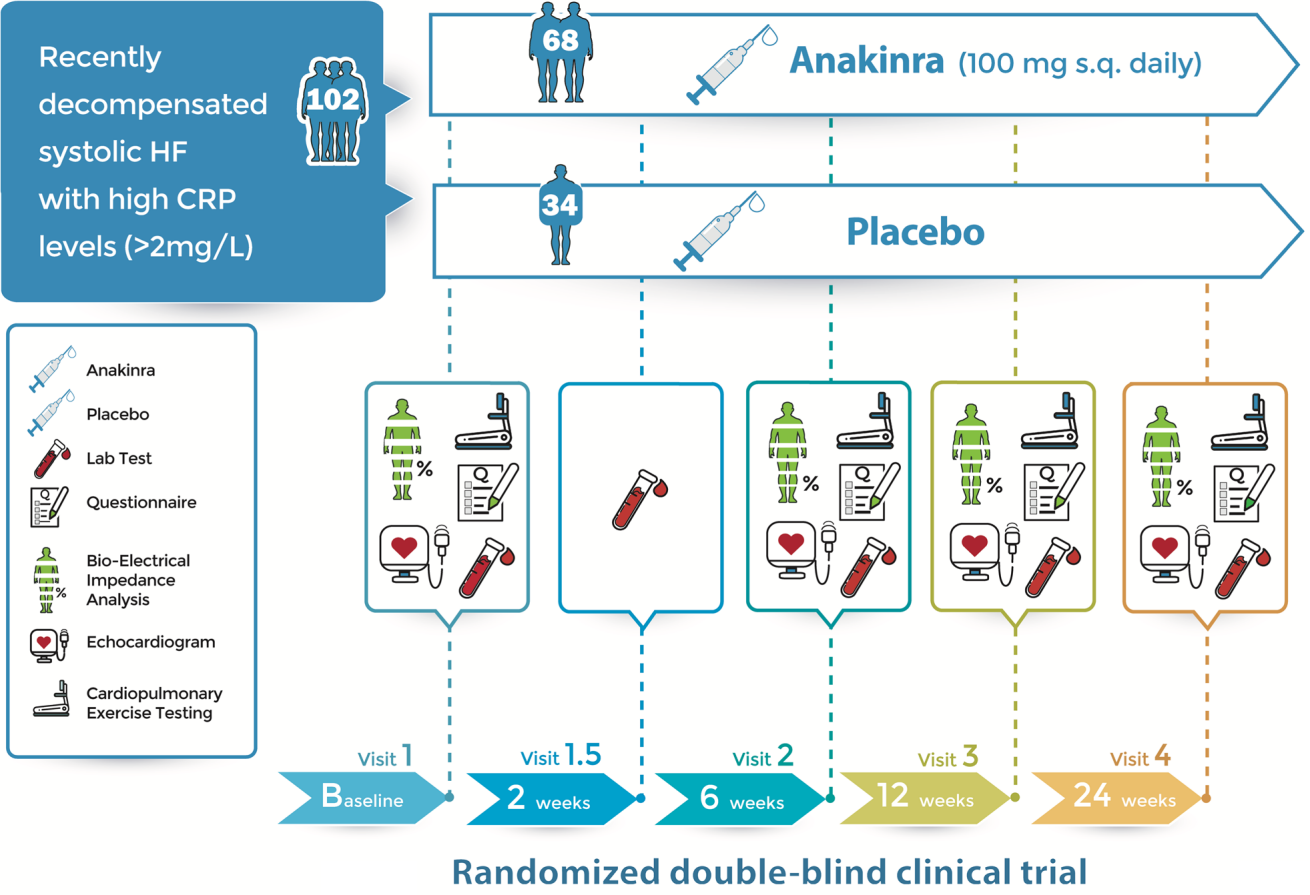
Schematic protocol of the REDHART2 study. Schematic protocol of the REDHART2 study to investigate the potential effects of anakinra in a randomized double-blind placebo-controlled clinical trial. Patients will be followed for 24 weeks. Lab tests, questionnaires, bio-electrical impedance analysis, echocardiogram and cardiopulmonary exercise testing will be done at baseline, 6, 12, and 24 weeks. Visit 1.5 (after 2 weeks) is considered a safety follow-up assessment

The REDHART2 study is a single-center, randomized, placebo-controlled, double-blinded, phase II clinical trial. Patients will be randomized in a 2:1 ratio to receive anakinra or placebo for 24 weeks. Figure 1 presents an overview of the study procedures.

Patients will undergo baseline clinical assessment, blood tests for biomarkers, transthoracic echocardiogram, body-composition analysis, QoL questionnaires, perceived functional capacity and physical activity questionnaires, and CPX. After completion of all baseline testing, patients that qualify for the study will be randomized and given a 14-day supply of anakinra or placebo. Clinical evaluations, blood tests, echocardiogram, body composition analysis, QoL questionnaires, perceived functional capacity and physical activity questionnaires, and CPX will be repeated at 6 ± 1 weeks, 12 ± 1 weeks, and 24 ± 2 weeks. A clinical evaluation will also be completed at 14 ± 3 days to evaluate preliminary tolerability of the treatment and measure a complete blood cell count with differential and comprehensive metabolic panels (Fig. 1). Additional supply of anakinra or placebo will be given at each visit.

### Screening and enrollment

Adult patients with acute decompensated HFrEF (left ventricular EF ≤ 40%) will be eligible for this study within 14 days of hospital discharge if they have elevated levels of high sensitivity CRP (hsCRP > 2 mg/L) and meet the remaining inclusion criteria (Table 1). Patients will be excluded if the primary diagnosis for admission is not acute decompensated HF or if they have any contraindications against anakinra use (e.g., pregnancy, neutropenia, dialysis, acute or chronic infections), or have any concomitant medications or conditions that could affect the inflammatory response [e.g., recent infection with severe acute respiratory syndrome coronavirus 2 (SARS-CoV-2), cancer, autoimmune diseases] (Table 1).

**Table 1:** Inclusion and exclusion criteria of REDHART2 study

Inclusion criteria	
Age ≥ 21 years old	
LVEF ≤ 40% in the last 12 months with any imaging modality	
Primary diagnosis for hospitalization admission is decompensated heart failure with **both** conditions: 1.Dyspnea or respiratory distress or tachypnea at rest or with minimal exertion 2.Evidence of elevated cardiac filling pressure or pulmonary congestion (**one** of the following met): •Pulmonary congestion/edema at physical exam OR chest X-Ray •Plasma BNP levels ≥ 200 pg/mL OR NTproBNP ≥ 600 pg/mL •Invasive measurement of LVEDP > 18 mmHg OR PA occluding pressure (wedge) > 16 mmHg	
Clinically stable, euvolemic, and meets standard criteria for hospital discharge as documented by **all 3** of the conditions listed below: 1.Absence of dyspnea or pulmonary congestion/distress at rest 2.Absence of pitting edema in the lower extremities, or in any other region 3.Stable hemodynamic parameters (blood pressure, heart rate)	
Willing and able to comply with the protocol (i.e., self-administration, exercise test, screening CRP)	
CRP > 0.3 mg/dL or hsCRP > 2 mg/L	
Exclusion criteria	
The primary diagnosis for admission is NOT decompensated heart failure (i.e., acute coronary syndromes, hypertensive urgency/emergency, tachy- or brady-arrhythmias)	
Concomitant comorbidities that would interfere with the execution or interpretation of the study (i.e., uncontrolled hypertension, orthostatic hypotension, tachy- or brady-arrhythmias, acute or chronic pulmonary disease, or neuromuscular disorders affecting respiration, peak respiratory exchange ratio (VCO_2_/VO_2_) < 1.0, or with angina, abnormal blood pressure or heart rate response, or ECG changes suggestive of coronary ischemia that limit maximum exertion during CPX obtained during the baseline testing	
Cardiac resynchronization therapy (CRT) during index hospitalization, or planned CRT or valvular heart surgery within the following 6 months	
Previous or planned implantation of left ventricular assist devices or heart transplant	
Chronic use of intravenous inotropes	
Recent (< 14 days) use of immunosuppressive or anti-inflammatory drugs (including oral corticosteroids at a dose of prednisone equivalent of 0.5 mg/kg/day but not including inhaled or low dose oral corticosteroids or oral NSAIDs)	
Chronic inflammatory disorder (including but not limited to rheumatoid arthritis, systemic lupus erythematosus)	
Active infection (of any type), including chronic/recurrent infectious disease (i.e., HBV, HCV, and HIV/AIDS)—but excluding HCV + with undetectable plasma RNA	
Current malignancy (excluding carcinoma in situ [any location] or localized non-melanoma skin cancer) receiving targeted therapy	
Any comorbidity limiting survival or ability to complete the study	
Evidence of COVID-19 within the last 60 days or recent (21 days) exposure to close personal contact	
Stage V kidney disease (eGFR < 15 mL/min/1.73m^2^) or on renal-replacement therapy	
Neutropenia (< 1500/mm^3^ or < 1000/mm^3^ in African American patients)	
Pregnancy	
Hypersensitivity to Kineret (anakinra) or to *E. coli*-derived products	

*BNP* brain natriuretic peptide, *CRP C*-reactive protein, *HBV* hepatitis B virus, *HCV* hepatitis C virus, *HIV* human immunodeficiency virus, *hsCRP* high sensitive C-reactive protein, *eGFR* estimated glomerular filtration rate, *LVEDP* left ventricular end diastolic pressurev, *LVEF* left ventricular ejection fraction, *NSAIDs* non-steroidal anti-inflammatory drugs, *PA* pulmonary artery, *RNA* ribonucleic acid, *VCO2/VO2* carbon dioxide production/oxygen consumption ratio

### Randomization

Randomization will be handled by the investigational pharmacy using a dedicated randomization algorithm. Patients and physicians who involve in the study treatment will be blinded to the allocation status.

### Investigational treatment

Anakinra (100 mg) or placebo (vehicle) dispensed in small syringes (0.67 mL) will be provided to the patient for daily subcutaneous injection. The syringes for anakinra or placebo have been purchased from the supplier (Swedish Orphan Biovitrum, Stockholm, Sweden) to ensure that they are indistinguishable from each other. Patients will also receive instruction from the investigators regarding self-injection technique, storage, and disposal.

### Data collection

#### Cardiopulmonary exercise testing (CPX)

CPX will be performed at baseline, and after 6 ± 1, 12 ± 2, and 24 ± 2 weeks. A physician-supervised maximal exercise test will be administered using a metabolic cart (UltimaCardioO2, MGC Diagnostics, Saint Paul, MN) that is interfaced with a conservative ramping treadmill protocol [24–26]. The highest 10-s average value for VO_2_ during the final 30 s defines peak VO_2_ in mLO_2_ kg^−1^ min^−1^ [27]. Peak respiratory exchange ratio (RER) will be used for determining subject effort. A peak RER ≥ 1.10 is a well-accepted criterion for maximal effort and a peak RER ≥ 1.0 is considered a minimal acceptable threshold. Subjects with RER < 1.0, indicating submaximal effort, or with angina, abnormal blood pressure or heart rate response, or ECG changes suggestive of coronary ischemia, at time of initial CPX will be excluded.

#### Doppler echocardiography

Transthoracic Doppler echocardiography will be performed at baseline, 6 ± 1, 12 ± 2, and 24 ± 2 weeks. All measurements will be performed by 2 operators blinded to treatment allocations.

#### Bioelectrical impedance analysis

Bioelectrical Impedance Analysis (BIA) will be conducted at baseline, and after 6 ± 1, 12 ± 2, and 24 ± 2 weeks. Body composition (i.e., water, lean mass, and fat) will be determined using a Quantum IV Body Composition Analyzer (RJL Systems, Inc., Clinton Township, MI).

#### Questionnaires

Subjects will complete the Kansas City Cardiomyopathy Questionnaire (KCCQ),[28] the Duke Activity Status Index (DASI) questionnaire,[29] International Physical Activity Questionnaire—Short Form (IPAQ-SF) [30], and the Patient Health Questionnaire-9 (PHQ-9) [31] at baseline and after 6 ± 1, 12 ± 2, and 24 ± 2 weeks. The KCCQ is a 20-question graded questionnaire that has been extensively validated to measure impairment in QoL in patients with HF. The DASI is a twelve-item “yes/no” questionnaire that allows for the calculation of perceived functional capacity. The IPAQ-SF is a 7-item questionnaire that allows the estimation of daily physical activity. Lastly, subjects will also be asked to complete the PHQ-9, a nine-question standardized assessment of depression and anxiety which has been validated in patients with HF.

#### Biomarkers

Blood will be collected at each visit (baseline, 6 ± 1, 12 ± 2, and 24 ± 2 weeks) and analyzed for a panel of complete blood cell count with differential, basic metabolic panel, hsCRP, and N-terminal pro-brain natriuretic peptide (NTproBNP). To maintain allocation concealment, the investigators will also be blinded to hsCRP levels throughout follow-up, which may be affected by treatment.

#### Clinical events

Clinical events of interest will include death (both cardiac and non-cardiac), hospitalizations (for HF, for other cardiac causes not related to HF, or for non-cardiac reasons), worsening of HF as an outpatient, nonfatal myocardial infarction, unstable angina, urgent or unplanned revascularization, tachy- or brady-arrhythmias leading to a new hospitalization or prolongation of hospital stay, acute kidney injury, acute respiratory failure, sepsis or other serious infection, acute ischemic stroke, acute hemorrhagic stroke, or acute stroke (indeterminate) (Table 2).

**Table 2:** Adjudicated clinical events in REDHART2 study

Death	
Cardiac death (in which a direct cause attributable to cardiac disease is present)	
-Sudden cardiac death (in which cardiac death occurred out of the hospital and suddenly; or in the hospital due to ventricular arrhythmias unrelated to other concomitant cardiac conditions) -Non-cardiac death (in which the event of death is considered not to be a direct consequence of cardiac disease)	
Hospitalization for any cause	
Hospitalization for heart failure (in which the primary diagnosis for hospitalization is decompensated heart failure established as the finding at admission of all 2 conditions listed: a. Dyspnea or respiratory distress or tachypnea at rest or with minimal exertion; b. Evidence of elevated cardiac filling pressure or pulmonary congestion (at least one of the conditions must be met: pulmonary congestion/edema at physical exam OR chest X-ray; plasma BNP levels ≥ 200 pg/mL; or invasive measurement of left ventricular end-diastolic pressure > 18 mmHg OR pulmonary artery occluding pressure (wedge) > 16 mmHg)	
Outpatient worsening of heart failure (defined as the need for intravenous diuretic treatment or need for increase in oral diuretic dose, or new prescription for first or add-on diuretic)	
Acute myocardial infarction, as defined by the WHO consensus statement 4th edition	
Unstable angina, or need for coronary revascularization	
Cardiac tachy-or brady-arrhythmias leading to a new hospitalization or to prolongation of hospital stay	
Acute renal failure (defined as an increase in plasma creatinine levels of 50% or 0.5 mg/L)	
Acute respiratory failure (not due to heart failure)	
Sepsis or other serious infection requiring antibiotic therapy	
Acute stroke	

Adjudication of these events will be performed by an ad hoc committee blinded to treatment allocation and the adjudication.

### Primary outcomes

The primary analysis of REDHART2 will compare the effects of IL-1 blockade with anakinra versus placebo on peak VO_2_ (in mLO_2_ kg^−1^ min^−1^) during CPX after 24 weeks of treatment.

### Secondary endpoints

A composite of cardiac death and re-hospitalization for HF within the first 6 months of hospitalization will serve as the clinical endpoint of interest for the proposed study. Cardiac death is defined as death in which a direct cause attributable to cardiac disease is present. Re-hospitalization for HF is defined as hospitalization in which the primary diagnosis is decompensated HF established as the finding at admission of both conditions listed: (1) dyspnea or respiratory distress or tachypnea at rest or with minimal exertion; and (2) evidence of elevated cardiac filling pressure or pulmonary congestion (defined as congestion/edema at physical exam or one of the following: congestion at chest X-ray, plasma BNP levels ≥ 200 pg/mL, invasive measurement of left ventricular end-diastolic pressure > 18 mmHg, or pulmonary artery occluding pressure (wedge) > 16 mmHg.

Exploratory endpoints include (1) additional CPX parameters: changes in peak VO_2_ at earlier endpoints (6 and 12 weeks), and changes in the VE/VCO_2_ slope, OUES, VO_2_ at VAT, and peak O_2_ pulse at 6, 12, and 24 weeks; (2) echocardiography parameters: changes in left ventricular volumes, diastolic function, right and left ventricular systolic function at 6, 12 at 24 weeks; (3) non-invasive hemodynamics: changes in ventriculo-arterial coupling at 6, 12 and at 24 weeks; (4) biomarkers: changes in hs-CRP and NTproBNP at 6, 12 and at 24 weeks; and (5) QoL assessment changes in the DASI and KCCQ, at 6, 12 and at 24 weeks.

### Specification of safety parameters

Safety parameters will include data collected at baseline and each visit, including physical examination, laboratory results, and functional and imaging tests. Disease-related safety data (HF-related) will be assessed, including changes in symptoms (or new symptoms), functional capacity, vital signs (including weight), renal function, or any significant changes in medications.

A complete blood cell count will be measured at each visit to exclude unusual cases of anakinra-related neutropenia (absolute neutrophil count [ANC] < 1000/mm^3^), for which suspension of active treatment will be considered until return to a value of ANC > 1800/mm^3^ (or > 1000/mm^3^ if patient is African American).

Adverse events (AEs) are defined as untoward medical occurrence in a patient administered a pharmaceutical product considered to be causally related to the study treatment or research conduct. Serious AEs (SAEs) are defined as any adverse event/experience occurring between patient baseline assessment and the final study visit those results in life threatening events, requiring hospitalization, persistent or significant disability or incapacity and is unexpected or not consistent with the natural history of the disease. All unexpected SAEs will be promptly (within 24 h) reported to the Virginia Commonwealth University Institutional Review Board (IRB) and data and safety monitoring board (DSMB).

### The data and safety monitoring board

The DSMB is composed of a coordinator and 5 voting members. The minutes from each meeting will be distributed to the board members, National Heart Lung Blood Institute (NHLBI) program officer, and to the IRB. A brief conclusive statement addressing whether the study should continue as planned will be provided to the investigators and the IRB every 6 months.

### Alternate consent group

Any potential participants who are eligible to enroll in the study but decline to participate will be offered an alternate consent process that allows the research team to access the electronic health record for 6 months after the enrollment to ascertain vital status and any HF hospitalizations during follow-up. This alternate consent group will provide an estimate of the event rate in a group free of research interventions.

### Statistical analysis

#### Demographics and baseline characteristics

Descriptive summaries of continuous measurements will be reported as median and interquartile ranges due to potential deviation from Gaussian distribution. Descriptive summaries of categorical measurements consist of frequencies, proportions, and 95% confidence intervals, when applicable.

#### Analysis

All analyses will be conducted after database locking once all data has been gathered and electronically captured. All analyses will be based on the intention-to-treat principle (i.e., analyzing groups as randomized and including all patients with outcome data available). The difference in interval changes in peak VO_2_ at 24 weeks between the anakinra versus placebo groups will be compared using random-effect analysis of variance for repeated measures to analyze the effects of treatment within each group and the effect of time x group allocation. Unadjusted p-values will be reported throughout, with statistical significance for the primary endpoint set at the 2-tailed alpha 0.05 level. To evaluate the group differences in the secondary endpoints, data will be compared across all groups using the random-effect analysis of variance for repeated measures as indicated above for paired analyses or using Chi-squared testing for event rates. The Statistical Package for Social Sciences software version 25 (IBM, New York, NY) will be used.

#### Sample size considerations

The sample size for this pilot study is calculated according to the primary endpoint of difference in interval change in peak VO_2_ at 24 weeks between anakinra and placebo. Given an expected average peak VO_2_ of 15 mLO_2_ kg^−1^ min^−1^ for HF patients, 68 subjects randomized to anakinra, and 34 subjects randomized to placebo (2:1 randomization) would provide approximately > 95% power to detect a difference of 1.6 mL O_2_ kg^−1^ min^−1^ (standard deviation of 1.7–2.0 mLO_2_ kg^−1^ min^−1^) in peak VO_2_ on top of placebo. A conservative estimate of 20% loss to follow-up or withdrawal would retain > 90% power (Table 3).

**Table 3:** Power estimate for 102 subjects based on peak VO_2_

	Peak VO_2_	Effect size of treatment on top of placebo				
	mL•kg^−1^• min^−1^	+ 1.2	+ 1.4	+ 1.6	+ 2.4	+ 3.2
Standard deviation	2.3	0.70	0.83	0.91	> 0.99	> 0.99
	2.0	0.82	0.92	0.97	> 0.99	> 0.99
	1.7	0.92	0.98	0.99	> 0.99	> 0.99
	1.4	0.98	0.99	> 0.99	> 0.99	> 0.99

Power estimation based on treatment effect of anakinra on the peak VO_2_ on the top of placebo for 102 subjects

## Discussion

The rationale for IL-1 blockade with anakinra in HF stems from the following evidence: (1) direct cardio-depressant effects of IL-1 in cardiac cells and animal models [4, 32, 33]; (2) reduced adverse cardiac remodeling HF with IL-1 blockers in animal models of AMI [15, 16, 34–36]; (3) reduced incidence of HF in patients with ST-segment elevation AMI [18, 19, 37]; 4) enhanced IL-1 activity in patients with HF [4]; (5) quenching of the acute inflammatory response in patients with acute decompensated HF [38]; and (6) favorable signals for improved cardiorespiratory fitness in pilot studies including patients with stable HFrEF, stable HF with preserved left ventricular ejection fraction (LVEF) (HFpEF), and in patients with recently decompensated systolic HF [23, 39, 40].

In the pilot REDHART trial, 60 patients with recently decompensated HFrEF were randomly assigned 1:1:1 to anakinra for 2 weeks, anakinra for 12 weeks, or placebo [23]. No significant differences in peak VO_2_ were seen with anakinra, or placebo, at 2 weeks. At 4 and 12 weeks, the patients treated with anakinra for 12 weeks showed an improvement in peak VO_2_ compared with baseline, whereas those treated with anakinra for 2 weeks or those treated with placebo did not improve peak VO_2_ [41]. In this trial, we expect that the increased sample size and prolonged treatment duration of the REHDART2 trial will provide sufficient power for an assessment of the treatment effect of IL-1 blockade on peak VO_2_. Furthermore, the REDHART2 trial will also employ a more stringent definition for “reduced” ejection fraction (LVEF ≤ 40%) as compared with the original pilot REDHART trial (LVEF ≤ 50%).

In the large multicenter CANTOS trial, treatment with canakinumab, IL-1β blocking antibody, reduced HF-related adverse events in patients with prior MI [20]. In a small single-center sub-study of the CANTOS trial in 15 patients with pre-existing HFrEF, canakinumab significantly increased peak VO_2_ at 3 months compared to placebo (median interval change of + 1.6 [from − 0.4 to + 3.4] mL kg^−1^ min^−1^; *P* = 0.026 between group changes) [40].

The limitations of the REDHART2 study should be discussed. This a single-center phase II clinical trial with a small sample size and relative short follow up period. While this is a limitation, the expertise required for the use of IL-1 blockers available at our center and the ability to standardize procedures related to peak VO_2_ measurement make this also a potential advantage. Furthermore, despite the small sample size, the study has a sufficient power for the primary endpoint. The study may not be powered to detect differences in clinical outcome, such as death or HF hospitalization, and while it may provide an estimate for the potential effect size on HF outcomes, these estimates need to be interpreted with caution. Moreover, sodium glucose cotransporter inhibitors received FDA approval during the conduct of this trial and have become part of guideline-directed medical therapy only more recently [42]. The resultant change in background therapy may increase the heterogeneity of the data. However, we hope to minimize the impact of this change—along with other potential evolutions in background therapy—through the ongoing process of randomized allocation. Finally, this study is being conducted during the COVID-19 pandemic, which has disrupted routine healthcare practices and may have altered the rates of hospitalization for many HF patients.

In conclusion, the REDHART2 study will determine whether IL-1 blockade with anakinra for 24 weeks will improve cardiorespiratory fitness measured with CPX, namely peak VO_2_, in patients with recently decompensated HFrEF.

## Data Availability

A simplified and fully de-identified database will be made available for sharing in accordance with requirements for NHLBI data repository datasets and associated documentation for submission to the Biological Specimen and Data Repository Information Coordinating Center (BioLINCC) and the NHLBI Policy for Data Sharing from Clinical Trials and Epidemiological Studies, and in accordance with the Guidelines for NHLBI Data Set Preparation, within 3 years of completion of the study.

## Abbreviations

AE: Adverse event
ANC: Absolute neutrophil count
AMI: Acute myocardial infarction
BIA: Bio-electrical impedance analysis
CANTOS: Canakinumab anti-inflammatory thrombosis outcome study
CPX: Cardiopulmonary exercise test
CRF: Cardiorespiratory fitness
DASI: Duke activity status index
DSMB: Data safety and monitoring board
Ea: Estimates of arterial elastance
Ees: End-systolic elastance
HF: Heart failure
HFpEF: Heart failure with preserved ejection fraction
HFrEF: Heart failure with reduced ejection fraction
hsCRP: High sensitive C-reactive protein
IL: Interleukin
IPAQ-SF: International physical activity questionnaire—short form
IRB: Institutional review board
KCCQ: Kansas city cardiomyopathy questionnaire
LVEF: Left ventricular ejection fraction
NHLBI: National heart lung blood institute
NLRP3: NOD-, LRR- and pyrin domain-containing protein 3
NT-proBNP: N-terminal pro brain natriuretic peptide
PHQ-9: Patient health questionnaire-9
OUES: Oxygen uptake efficiency slope
QoL: Quality of life
REDHART: REcently decompensated heart failure anakinra response trial
RER: Respiratory exchange ratio
SAE: Severe adverse event
VAT: Ventilatory anaerobic threshold
